# Clinical Characteristics, Identification, and Treatment of Lichen Planus: A Case Report

**DOI:** 10.7759/cureus.65712

**Published:** 2024-07-29

**Authors:** Tikeshwari Gurav, Aman Thakare, Priya Shinde, Mahesh Chavan, Kushal Taori, Vikrant Jadhav

**Affiliations:** 1 Oral Medicine and Radiology, Sharad Pawar Dental College and Hospital, Datta Meghe Institute of Higher Education and Research, Wardha, IND; 2 Orthodontics and Dentofacial Orthopaedics, Sharad Pawar Dental College and Hospital, Datta Meghe Institute of Higher Education and Research, Wardha, IND

**Keywords:** ointment clobetasol propionate, wickham's striae, lichenoid reaction, systemic disease, oral lichen planus

## Abstract

Women are primarily affected by lichen planus, a chronic autoimmune skin and mucosal disorder in their 40s to 60s. Medication, systemic disorders, and mental stress are some of the factors that can cause it, though the precise cause is still unknown. Middle-aged females are the main victims of the disease; children are rarely affected. Oral lichen planus (OLP) is less common in atrophic and erosive forms. There is disagreement over whether OLP causes cancer; therefore physicians need to keep a close watch for any intraoral lichenoid lesions, and patients with OLP are advised to follow up regularly. This case report details a 52-year-old woman's right buccal mucosa and tongue affected by reticular lichen planus.

## Introduction

Lichen means "moss," and planus means "no raised surface" in Greek. Erasmus Wilson coined the term lichen planus for the first time in 1869 [1, 2]. The primary target of lichen planus, a chronic skin and mucosal illness, is the squamous epithelium layer of the body. It primarily affects women, with an incidence rate of 1.3% [3]. Clinically, oral lichen planus (OLP) is classified into four forms: reticular, atrophic, erosive, or bullous forms [4]. The most common kinds are reticular and erosive, which appear as papules and plaques with an erythematous border and white, overlapping keratotic lines; this condition is referred to as Wickham striae [5].

The lesions are located in the lateral borders of the tongue, gingiva, lips, and buccal mucosa (often bilaterally) [6]. Numerous factors, including vitamin deficiencies, hypersensitivity to dental materials, genetics, drug reactions, graft-versus-host disease (GVHD), and infectious agents, have been suggested as potential causes of lichen planus, even though the exact cause is still unknown. Although the precise cause of lichen planus is unknown, damage to the epithelium by cell-mediated immunity is suggested by a T-lymphocyte infiltrate [7]. Even though the cluster of differentiation (CD-8) (cytotoxic) and CD-4 (helper) cells exist, only CD-8 cell activation can damage the basal epithelium [7].

## Case presentation

**Figure 1:**
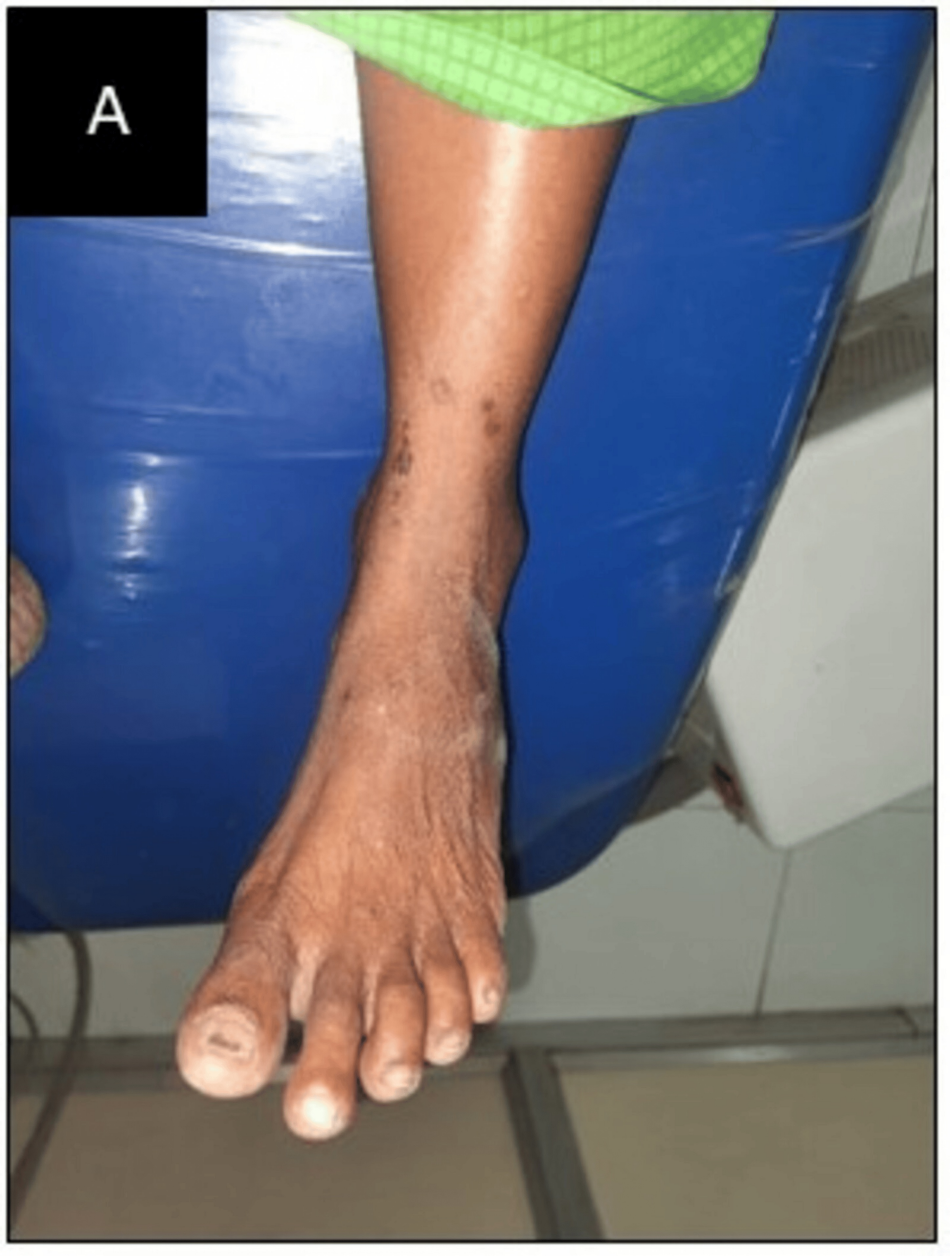
Papules on the leg

The main complaint of a 52-year-old woman who had been experiencing burning in her mouth for the past two months was brought to the attention of Oral Medicine and Radiology Department. The patient was alright one year back. Then she noticed discoloration on her left and right buccal mucosa and her tongue. She had started experiencing a burning sensation in her mouth for two months. She experienced burning sensations only when eating spicy food. The burning sensation lasted 10-15 min after having food and then relieved on its own. There is no relevant medical history or habit history. The patient had a history of family stress. Sleep was irregular and disturbed. The patient was alert, cooperative, and well-oriented, and her vital signs were within normal ranges. Extraoral examinations were normal. On the upper and lower extremities, flap papules were present and the patient had a history of itching. Figure 1 depicts small, annular, and flat-topped papules on the leg.

**Figure 2:**
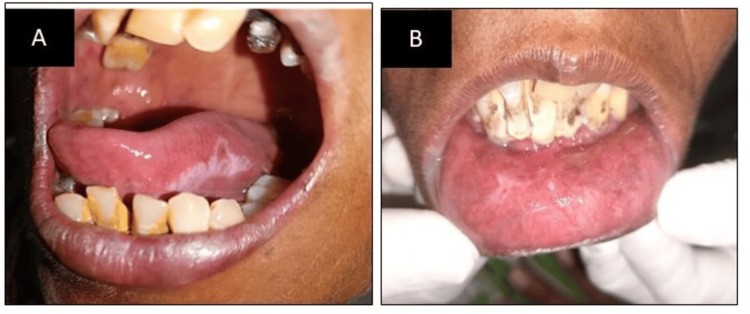
(A) Striations on the right lateral border of the tongue; (B) striations on the lower labial mucosa

Intraoral examination of the patient revealed white lines or striae (radiating Wickham’s striae) like lesions observed on the tongue's right lateral border (approximately 2x3cm) as shown in Figure 2A and on the lower labial mucosa as shown in Figure 2B.

On palpation, the intraoral lesions were non-tender and non-scrapable. Additionally, the mucosal surface was smooth and soft in consistency. Therefore, OLP was the tentative diagnosis. The differential diagnosis was lichenoid reaction and psoriasis. Soft, thread-like, white, or gray papules that radiate were arranged in a linear, annular, or retiform pattern characterizing the reticular rings, patches, and streaks on the buccal mucosa and, to a lesser extent, on the tongue, palate, and lips. In OLP, the "striae of Wickham" are the little raised white dots that frequently show up where the white lines intersect. In lichenoid reaction, lesions or ulcerations that are reticular, erythematous, erosive, and have a white streak resembling Wickham's striae of lichen planus were seen.

Blood investigations as shown in Table 1 (full blood count, clotting time, bleeding time, and blood sugar residue) were done and the hemoglobin level was found to be low (10.2 g/dl). The final diagnosis was lichen planus (reticular type). We conclude that the patient has lichen planus based on the history of sleep disturbances, the intraoral appearance of Wickham striae, characteristic features of the condition, and the results of our blood workup, which included a decreased hemoglobin level as one of the causes.

**Table 1:** Blood workup results.

Parameters	Patient value	Reference range
Hemoglobin	10.2 g/dl	11-16 g/dl for females; 15-18 g/dl for males
Neutrophils	64%	48 -75%
Lymphocytes	25%	20-40%
Eosinophil	4%	0-6%
Monocytes	7%	0-8%
Basophils	0%	0.5-1%
Bleeding time	1 min 0 sec	<6 min
Clotting time	5 min 0 sec	5-10 min
Plasma glucose (random)	99 mg/dl	<110 mg /dl

Treatment

The patient was counseled to reduce the stress levels. The immediate treatment included the application of ointment clobetasol propionate 0.05% (thrice a day for one week).

**Figure 3:**
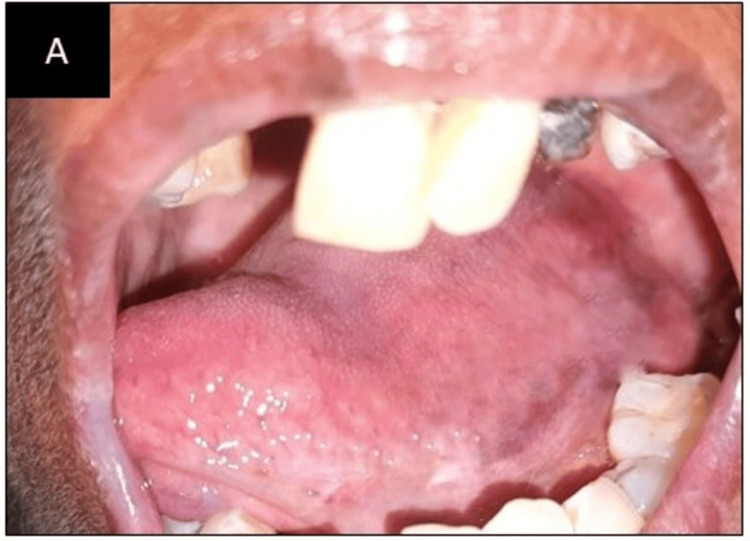
Post-treatment pictures after one month: (A) Right lateral border of the tongue.

Then, topical oral clobetasol 0.05% gel was combined with acitretin, which was first started at 10 mg per day for two weeks before being increased to 25 mg per day. The patient lost weight, became less fatigued, and the size of the oral lesions shrank. Because the side effects persisted, the dosage of acitretin was reduced to 10 mg per day for four weeks before it was discontinued. We administer acitretin for a brief period of time to minimize side effects. When taken long-term, common side effects include xerostomia, dry eyes, and hair loss. Vitamin A found in acitretin has anti-inflammatory and anti-proliferative properties. We administered oral prophylaxis and provided dental hygiene education to the patient. The patient was called after one month for a review. Figure 3A shows a complete reduction of the striations on the right lateral border of the tongue. Later, the patient was called for follow-up every month for six months. We monitored the patient for three months after the lesion fully healed, but the patient had not reported for follow-up after four months.

## Discussion

An autoimmune condition called lichen planus results in inflammation [8]. It is the most common non-infectious disease that affects adult patients' oral mucosa. Only 17% of patients with OLP lesions make a full recovery, even though 39% of the cases have been reported to experience remissions. Although the precise cause of this illness is unknown, stress, medication, dental fillings, immunity, and hypersensitivity reactions can all be factors in its pathogenesis. With an incidence ranging from 0.6% to 2.5%, oral lichen planus (OLP) is most common in women between the ages of 35 and 65 [9].

The body's mucous membranes and skin are the main organs impacted by this persistent inflammatory disease [10]. The most prevalent type of OLP is characterized by Wickham's striae with a reddish outline, the so-called reticular form [11]. The main areas of irritation are the lips (2%), tongue (14%), buccal mucosa (62%), and alveolar mucosa (19%). The percentage of occurrence of oral mucosa was verified in the case we presented by the right border of the tongue and the cheek mucosa [12].

T-cells, particularly CD8-positive ones, release two cytokines that damage the basement membrane: tumor necrosis factor-alpha (TNFα) and interleukin-12 (IL-12), which mediate OLP [13]. Patients with OLP may get additional skin lesions or lesions at different mucosal sites. The cutaneous lesions are violaceous, flat-topped, polygonal, and erythematous papules covered in a network of fine lines called Wickham striae [14]. The cutaneous lesions typically show up a few months after the oral lesions do.

In certain studies, OLP has been found to coexist with a recognized autoimmune disease (primary biliary cholangitis, Sjogren's syndrome, or systemic lupus erythematosus). This implies that autoimmune processes may be connected to at least some of the OLP lesions. Thus, the pathophysiology of OLP may involve the interdependent mechanisms between B- and T-lymphocytes. Treatment of this complicated pathology is difficult. The application of precision therapeutic strategies using monoclonal antibodies and B- and T-cell-specific inhibitors can be aided by the characterization of the B- and T-cell subpopulations.

Although there are many different ways to treat OLP, the first line of treatment is typically topical corticosteroids like 0.05% betamethasone and 0.10% triamcinolone acetonide ointment [15]. When retinoic acid receptors (RARs) and retinoid X receptors (RXRs) bind to nuclear receptors, the expression of genes involved in cell differentiation, proliferation, and apoptosis is modulated. Cell growth may return to normal as a result, and hyperproliferative circumstances may subside. While the illness is still actively present, the patient may be advised to maintain good oral hygiene. Due to the high teratogenic potential of acticin, women who are or will become pregnant should use effective contraception both during and for a considerable amount of time after the treatment ends. However, therapy for OLP needs to start immediately and should be checked on frequently because certain types of it have a high chance of turning into cancer [16]. Clinical scores to assess improvement in lichen planus are shown in Table 2.

**Table 2:** Clinical scores to assess improvement in lichen planus OLP: oral lichen planus

Follow up	Visual analog scale (VAS) score	Thongprasom sign scoring for OLP
First visit	8	3
Second visit	5	2
Third visit	1	1

When diagnosing and treating OLP, Aghbari et al. emphasize that clinicians must be cognizant of the associated systemic conditions and demographic factors [17]. Understanding the immunological mechanisms underlying OLP and proposing possible targets for therapeutic intervention are provided by Awad et al. [18]. In order to differentiate OLP from oral lichenoid reaction (OLR) and determine the best course of treatment, McQuaid et al. stress the significance of a comprehensive clinical and histopathological examination [19]. Kalaskar et al. support the effectiveness of topical corticosteroids in the treatment of OLP, but raise concerns about their possible side effects [20].

## Conclusions

An immune-mediated chronic condition, lichen planus usually affects the gingiva and buccal mucosa. After corticosteroids and immunomodulatory medications were administered, the patient showed positive clinical development. Medication improved the prognosis. When oral lichen planus is histologically confirmed, the clinician must advise the patient of the recurrent pattern and any symptoms of exacerbation. To determine the potential for malignancy, the clinician must also keep track of all intraoral lesions over an extended time.

## References

[1] Pauly G, Kashyap R, Kini R, Rao P, Bhandarkar G (2017). Reticular oral lichen planus: the intra-oral web - a case report. Gulhane Med J.

[2] Eisen D, Carrozzo M, Bagan Sebastian JV, Thongprasom K (2005). Number V oral lichen planus: clinical features and management. Oral Dis.

[3] Boorghani M, Gholizadeh N, Taghavi Zenouz A, Vatankhah M, Mehdipour M (2010). Oral lichen planus: clinical features, etiology, treatment and management; a review of literature. J Dent Res Dent Clin Dent Prospects.

[4] (2003). Burket’s Oral Medicine. Burket’s Oral Medicine. 10th ed. Hamilton: BC Decker Inc.

[5] Edwards PC, Kelsch R (2002). Oral lichen planus: clinical presentation and management. J Can Dent Assoc.

[6] Murti PR, Daftary DK, Bhonsle RB, Gupta PC, Mehta FS, Pindborg JJ (1986). Malignant potential of oral lichen planus: observations in 722 patients from India. J Oral Pathol.

[7] Alam F, Hamburger J (2001). Oral mucosal lichen planus in children. Int J Paediatr Dent.

[8] Vlad CS, Vlad DC, Popescu R (2020). Oral lichen planus - case report. Rom J Morphol Embryol.

[9] Usatine RP, Tinitigan M (2011). Diagnosis and treatment of lichen planus. Am Fam Physician.

[10] (2006). Shafer’s Textbook of Oral Pathology, 5th ed. Shafer’s Textbook of Oral Pathology. 6th ed.India: Elsevier, a Division of Reed Elsevier India Private Limited; 2009. p. 799-803.

[11] Ismail SB, Kumar SK, Zain RB (2007). Oral lichen planus and lichenoid reactions: etiopathogenesis, diagnosis, management and malignant transformation. J Oral Sci.

[12] McCartan BE (1995). Psychological factors associated with oral lichen planus. J Oral Pathol Med.

[13] Chaitanya NC, Chintada S, Kandi P, Kanikella S, Kammari A, Waghamare RS (2019). Zinc therapy in treatment of symptomatic oral lichen planus. Indian Dermatol Online J.

[14] Alsarraf A, Mehta K, Khzam N (2019). The gingival oral lichen planus: a periodontal-oral medicine approach. Case Rep Dent.

[15] Farhi D, Dupin N (2010). Pathophysiology, etiologic factors, and clinical management of oral lichen planus, part I: facts and controversies. Clin Dermatol.

[16] Scully C, Carrozzo M (2008). Oral mucosal disease: lichen planus. Br J Oral Maxillofac Surg.

[17] Aghbari SM, Zayed SO, Shaker OG, Abushouk AI (2019). Evaluating the role of tissue microRNA-27b as a diagnostic marker for oral lichen planus and possible correlation with CD8. J Oral Pathol Med.

[18] Awad AM, Kumar P, Ismail-Fitry MR, Jusoh S, Ab Aziz MF, Sazili AQ (2021). Green extraction of bioactive compounds from plant biomass and their application in meat as natural antioxidant. Antioxidants (Basel).

[19] McQuaid A, Sanatinia R, Farquharson L, Shah P, Quirk A, Baldwin DS, Crawford M (2021). Patient experience of lasting negative effects of psychological interventions for anxiety and depression in secondary mental health care services: a national cross-sectional study. BMC Psychiatry.

[20] Kalaskar AR, Bhowate RR, Kalaskar RR, Walde SR, Ramteke RD, Banode PP (2020). Efficacy of herbal interventions in oral lichen planus: a systematic review. Contemp Clin Dent.

